# Supplementary material for: Structural characterization of tin in toothpaste by dynamic nuclear polarization enhanced 119Sn solid-state NMR spectroscopy
Source: Nat Commun. 2023 Nov 16;14:7423. doi: 10.1038/s41467-023-42816-z (PMC10654397; doi:10.1038/s41467-023-42816-z)
Supplement: Supplementary file 1 — Supplementary information [file 41467_2023_42816_MOESM1_ESM.pdf]

Supplementary Material

Structural Characterization of Tin in Toothpaste By Dynamic Nuclear Polarization Enhanced ^{119}Sn Solid-State NMR Spectroscopy

Rick W. Dorn,[1,2] Scott L. Carnahan,[1,2] Chi-yuan Cheng,[3] Long Pan,[3] Zhigang Hao,[3] Aaron J. Rossini,[1,2*]*

[1]US Department of Energy, Ames National Laboratory, Ames, IA, USA, 50011.

[2]Iowa State University, Department of Chemistry, Ames, IA, USA, 50011.

[3]Colgate-Palmolive Company, Piscataway, NJ, USA 08855.

AUTHOR INFORMATION

Corresponding Author

*e-mail: zhigang_hao@colpal.com, phone: 732-878-6218.
*e-mail: arossini@iastate.edu, phone: 515-294-8952.

Table of Contents

	Page
**Supplementary Figures**	
**Supplementary Figure 1.** 2D $^{19}F\{^{119}Sn\}$ *J*-HMQC spectrum of SnF_4	S3
**Supplementary Figure 2.** 1D ^{19}F NMR spectra of SnF_2 and SnF_4	S4
**Supplementary Figure 3.** 2D $^{19}F\{^{119}Sn\}$ *J*-HMQC spectrum of SnF_2	S5
**Supplementary Figure 4.** 1D ^{19}F solution NMR spectra of the model toothpaste	S6
**Supplementary Figure 5.** 2D solution $^{19}F\{^{119}Sn\}$ *J*-HMQC spectrum of the model toothpaste	S7
**Supplementary Figure 6.** 1D room temperature ^{119}Sn NMR spectra	S8
**Supplementary Figure 7.** $^{1}H\rightarrow^{13}C$ CPMAS DNP enhancements	S10
**Supplementary Figure 8.** $^{1}H\rightarrow^{119}Sn$ CP-CPMG DNP enhancements	S11
**Supplementary Figure 9.** $^{1}H\rightarrow^{13}C$ and $^{1}H\rightarrow^{119}Sn$ CP-HETCOR spectra of preventative gel **1**	S12
**Supplementary Figure 10.** Mass spectra of SnF_2-glycerol complex	S13
**Supplementary Figure 11.** $^{1}H\rightarrow^{119}Sn$ CP-CPMG VOCS spectra of toothpaste samples	S14
**Supplementary Figure 12.** 2D ^{119}Sn aMAT spectrum of preventative gel **1**	S15
**Supplementary Figure 13.** 2D ^{119}Sn aMAT spectrum of toothpaste **2**	S16
**Supplementary Figure 14.** 2D ^{119}Sn aMAT spectrum of toothpaste **3**	S17
**Supplementary Figure 15.** 2D ^{119}Sn aMAT spectrum of toothpaste **4**	S18
**Supplementary Figure 16.** Effect of the number of echo trains during the acquisition of $^{1}H\rightarrow^{119}Sn$ CP-CPMG spectra	S20
**Supplementary Figure 17.** ^{119}Sn MAS NMR spectra of SnF_2 obtained with different MAS frequencies.	S21
**Supplementary Tables**	
**Supplementary Table 1.** DFT Calculated ^{119}Sn and ^{19}F NMR Parameters of SnF_2 and SnF_4	S2
**Supplementary Table 2.** Major toothpaste ingredients	S9
**Supplementary Table 3.** ^{119}Sn CP-CPMG fitting parameters	S19
**Supplementary Table 4.** Experimental NMR parameters	S22-S23

Supplementary Table 1. Periodic plane-wave GIPAW DFT calculated ^{119}Sn and ^{19}F NMR parameters of SnF_2 and SnF_4.

Species	Site/Nucleus	σ_{iso} (ppm)[a]	Ω (ppm)	κ
		^{119}Sn		
SnF_4	**Sn**F_6	3478.4	545.8	–1.0
SnF_2	**Sn**F_3	3517.5	661.5	–0.37
	**Sn**F_5	3594.3	593.4	–0.41
		^{19}F		
SnF_4	**1**	297	-	-
	**2**	271	-	-
SnF_2	**1**	168	-	-
	**2**	169	-	-
	**3**	180	-	-
	**4**	191	-	-

[a]Relative differences in isotropic shielding (σ_{iso}) are theoretically the same as relative differences in isotropic shift (δ_{iso}). A higher shielding value means that the chemical shift will be lower.

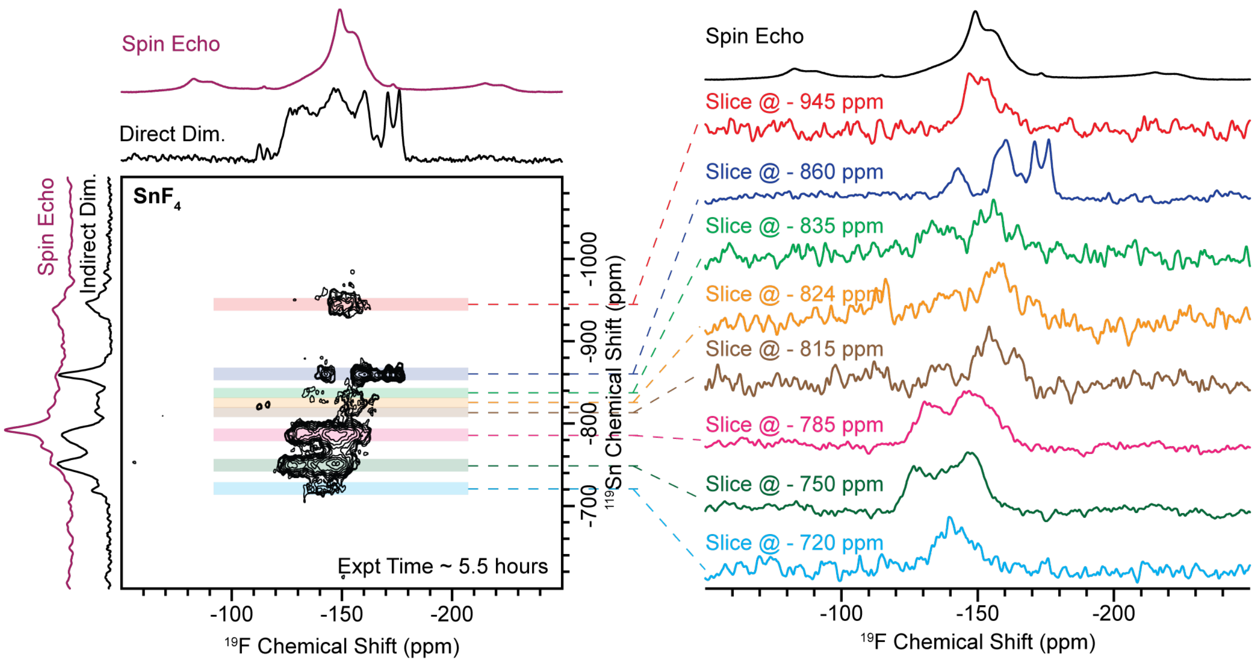

Supplementary Figure 1. (Left) 2D $^{19}F\{^{119}Sn\}$ *J*-HMQC solid-state NMR spectrum of SnF_4 recorded with 320 μs of total *J*-evolution and a 25 kHz MAS frequency. (Right) ^{119}Sn NMR spectra extracted from the rows of 2D $^{19}F\{^{119}Sn\}$ *J*-HMQC spectrum at the indicated ^{119}Sn chemical shifts. 1D spin echo NMR spectra are shown as purple traces above the projections of indirect and direct dimensions of the 2D NMR spectrum. The rows of the 2D spectrum (various colors) illustrate the ^{19}F chemical shifts at different ^{119}Sn chemical shift positions. Dashed lines indicate the ^{119}Sn chemical shifts and the corresponding rows showing the spectra (slice) extracted at each row.

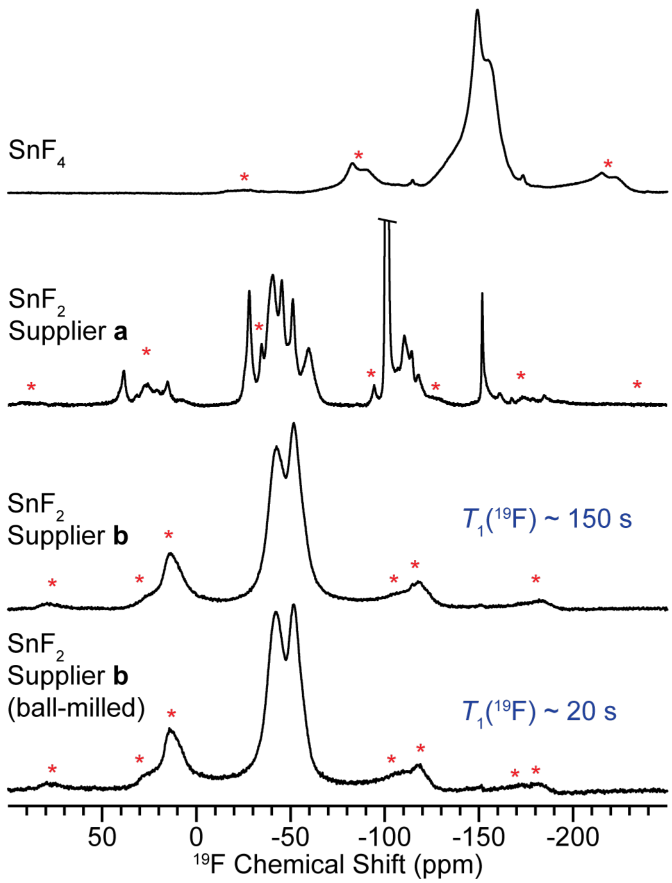

Supplementary Figure 2. Comparison of 1D ^{19}F solid-state NMR spectra of (upper to lower) SnF_4, SnF_2 from supplier **a**, and SnF_2 from supplier **b** (directly from the bottle or ball-milled). Asterisks (*) denote spinning sidebands. Spectra were recorded with an MAS frequency of 25 kHz.

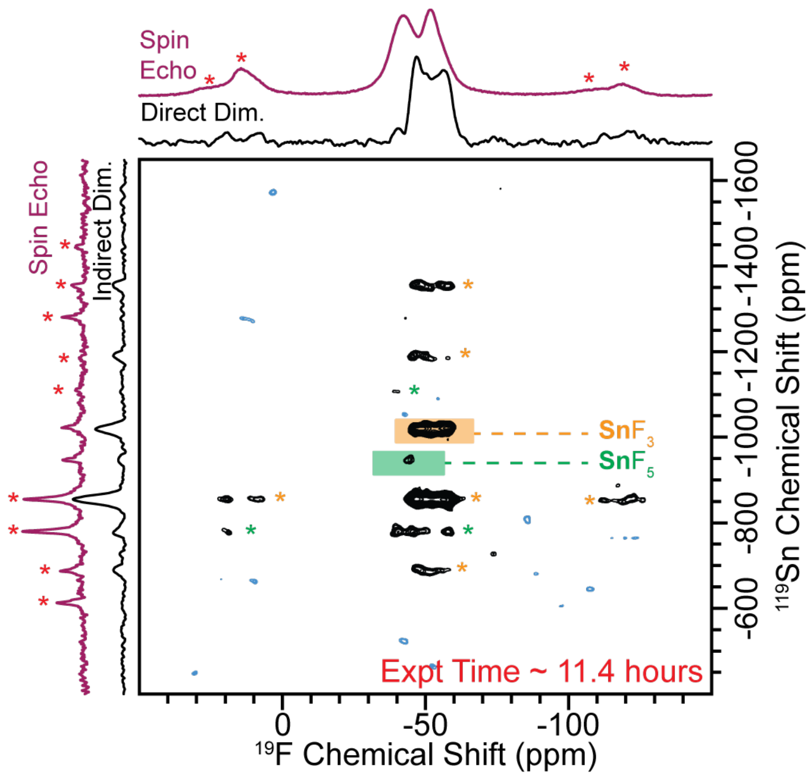

Supplementary Figure 3. 2D $^{19}F\{^{119}Sn\}$ *J*-HMQC solid-state NMR spectrum of ball-milled SnF_2 from supplier **b** recorded with a 25 kHz MAS frequency and 80 µs of total *J*-evolution. Asterisks (*) indicate spinning sidebands. The reduction in signal intensity for the SnF_5 site presumably arises from decreased values of $^1J(^{119}Sn-^{19}F)$ and/or reduced ^{19}F transverse relaxation times. 1D spin echo NMR spectra are shown as purple traces above the projections of indirect and direct dimensions of the 2D NMR spectrum. Orange and green boxes highlight isotropic ^{119}Sn NMR signals assigned to the SnF_3 and SnF_5 sites in the SnF2 crystal structure.

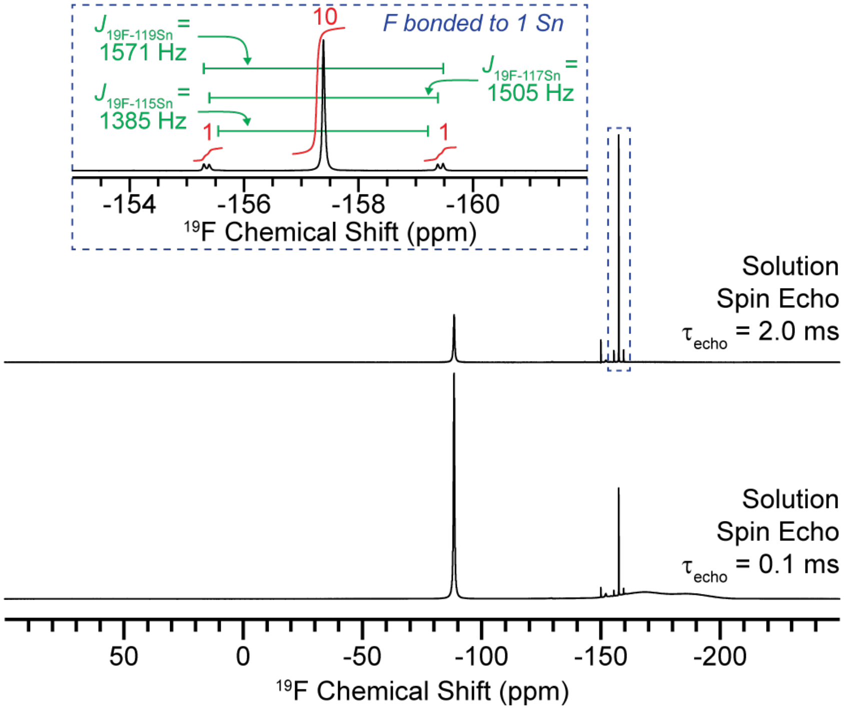

Supplementary Figure 4. 1D ^{19}F spin echo solution NMR spectra the model toothpaste recorded with a (upper) 2 ms or (lower) 0.1 ms echo period. The broad humps from *ca.* –150 to –200 ppm are background ^{19}F NMR signals from Teflon within the probe. The dashed lines indicate the expanded view showing ^{19}F NMR signals that correspond to F atoms which are covalently bound to a single Sn atom. The *J*-couplings to the different tin isotopes are indicated.

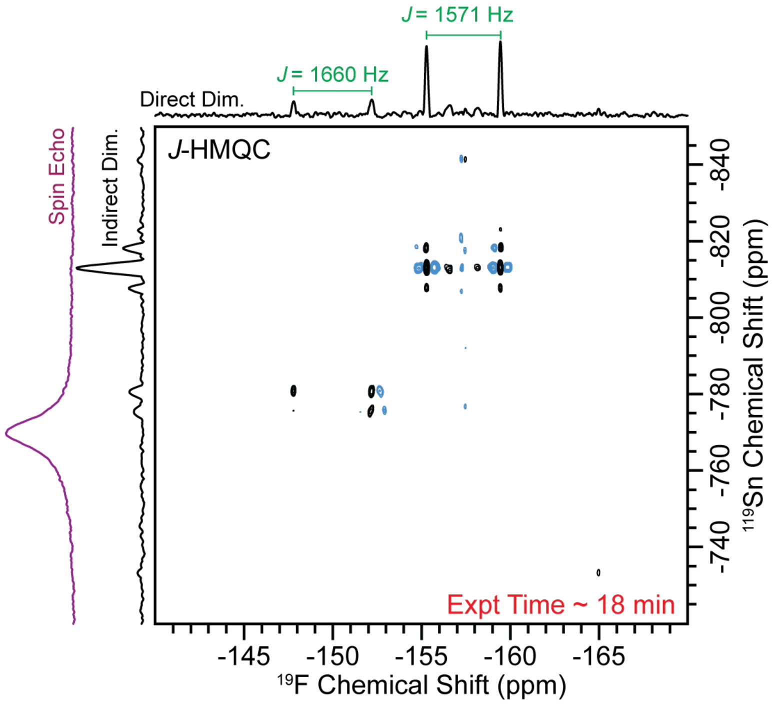

Supplementary Figure 5. 2D $^{19}F\{^{119}Sn\}$ *J*-HMQC solution NMR spectrum of the model toothpaste. Both sets of ^{119}Sn NMR signals exhibit multiple peaks because of truncation of the indirect dimension NMR signals. The 1D solution spin echo ^{119}Sn NMR spectrum is shown as a purple trace above the projection of the indirect dimension. ^{119}Sn-^{19}F one-bond *J*-couplings are indicated.

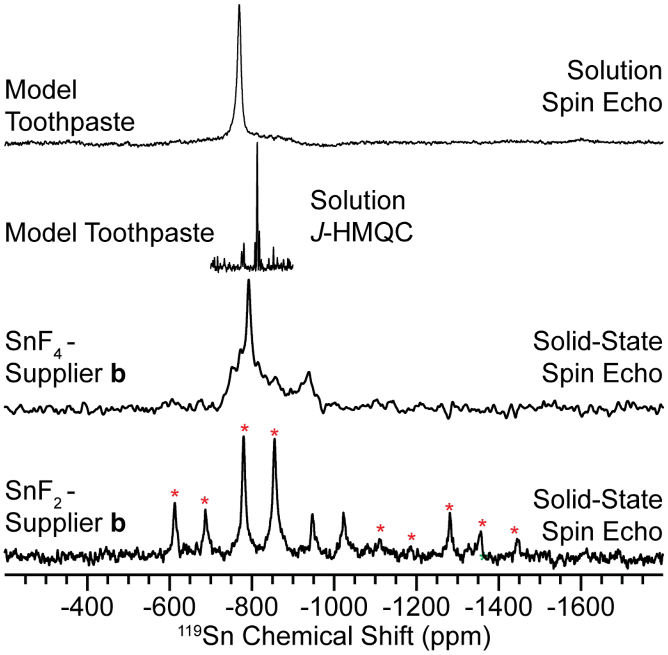

Supplementary Figure 6. Comparison of (upper two) ^{119}Sn spin echo and ^{19}F{^{119}Sn} *J*-HMQC solution NMR spectra of the model toothpaste with that of (lower two) ^{119}Sn spin echo solid-state NMR spectra of SnF_4 and SnF_2 (supplier **b**). Asterisks denote spinning sidebands.

Supplementary Table 2. Major ingredients in preventative gel **1** and toothpastes **1-4**.

Sample	Main Solvent	SnF_2 Stabilizer
Preventative gel **1**	Glycerin	No
Toothpaste **1**	Glycerin	No
Toothpaste **2**	Water, Sorbitol, Glycerin	No
Toothpaste **3**	Glycerin	No
Toothpaste **4**	Water, Sorbitol	$SnCl_2$

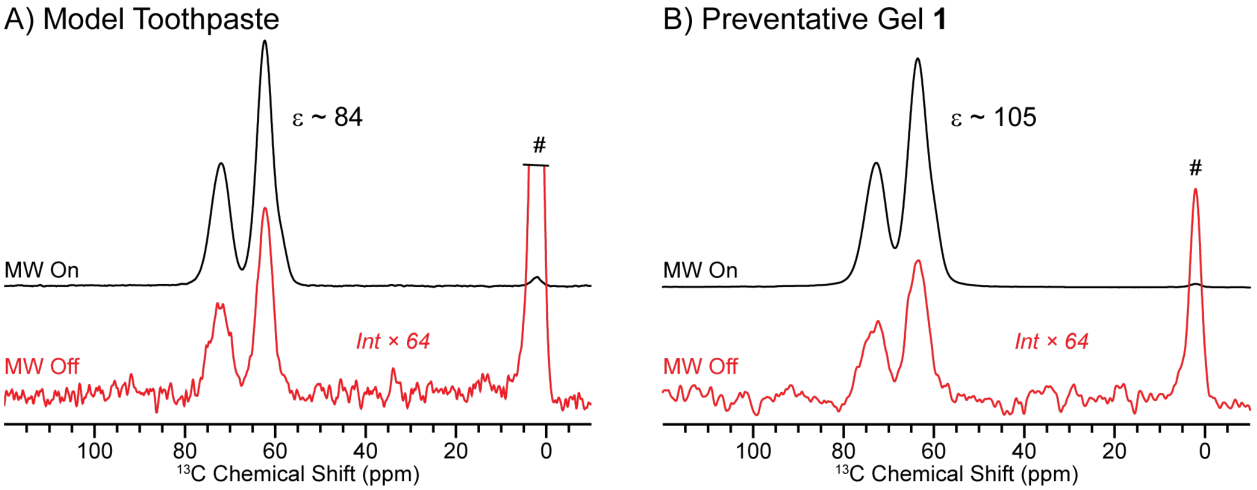

Supplementary Figure 7. $^{1}H \rightarrow ^{13}C$ CPMAS spectra of (A) the model toothpaste and (B) preventative gel **1** recorded (black) with or (red) without microwave (MW) irradiation of the electron spins. The DNP enhancements (ε) are given in the figure. The intensity of the spectra without microwave irradiation was increased by a factor 64.

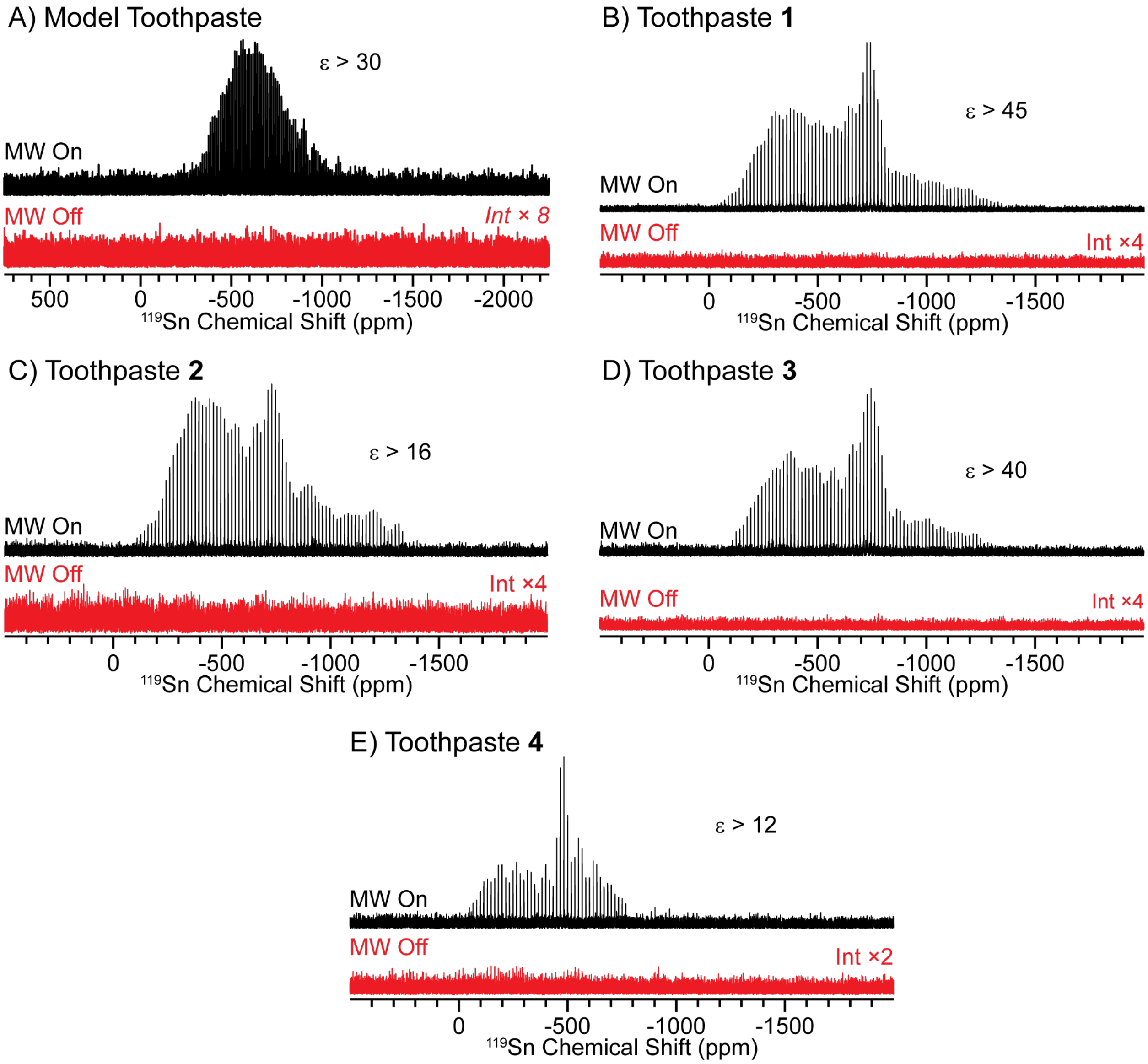

Supplementary Figure 8. $^{1}H \rightarrow ^{119}Sn$ CP-CPMG NMR spectra of the (A) model toothpaste and toothpastes (B) **1**, (C) **2**, (D) **3** and (E) **4** recorded (black) with or (red) without microwave (MW) irradiation of the electron spins. The estimated DNP enhancements (ε) are given in the figure.

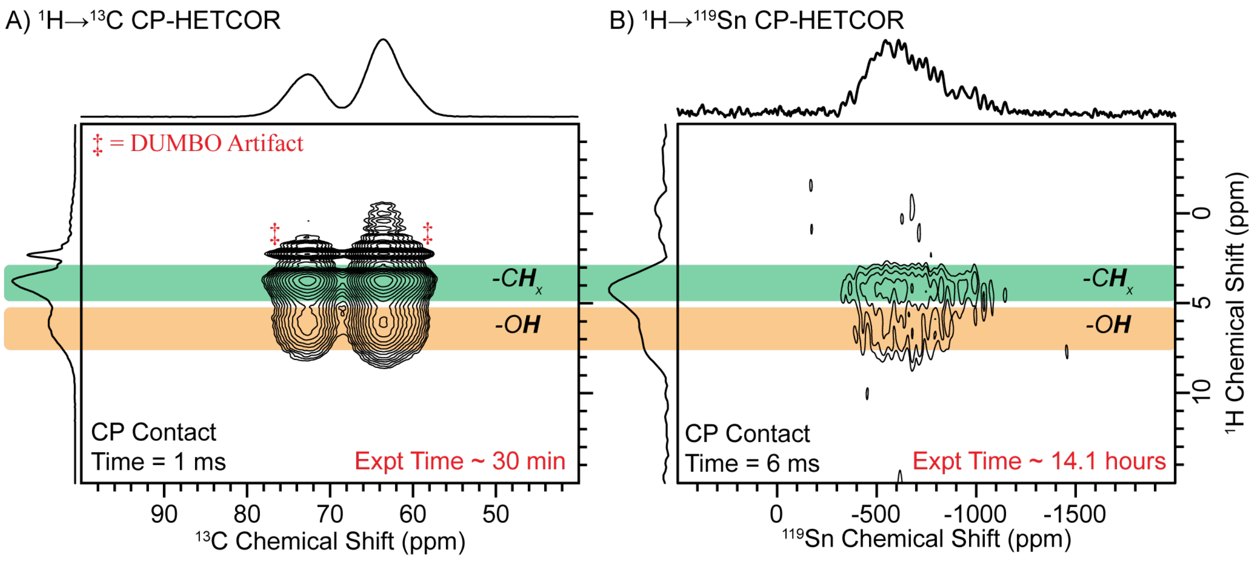

Supplementary Figure 9. DNP-enhanced 2D (A) $^{1}H\rightarrow^{13}C$ and (B) $^{1}H\rightarrow^{119}Sn$ CP-HETCOR NMR spectra of preventative gel **1** record with a 10 kHz MAS frequency, eDUMBO$_{1\text{-}22}$ ^{1}H homonuclear decoupling during the indirect acquisition of ^{1}H, and (B) CPMG detection of ^{119}Sn. Green boxes indicate the ^{1}H chemical shifts associated with CH protons of glycerol and orange boxes indicate ^{1}H chemical shifts associated with OH protons.

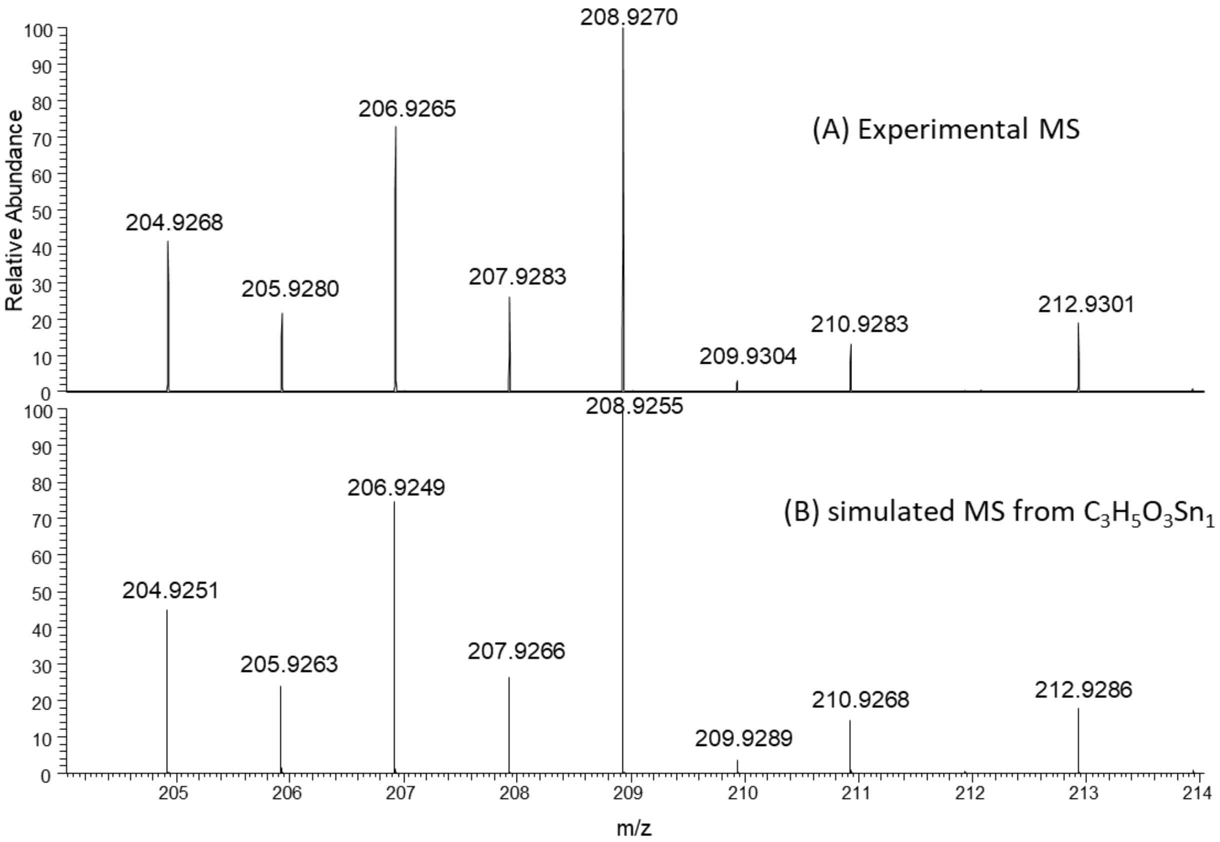

Supplementary Figure 10. Mass spectra of a Sn^{2+}-glycerol complex ($C_3H_5O_3Sn_1$). When a solution of 0.45 wt.% SnF_2 in glycerol was made and heated to 60 °C, the Sn^{2+}-glycerol complex could be detected with direct injection mode on a liquid chromatography-high resolution mass spectrometer. The delivery solvent was 50% methanol-water.

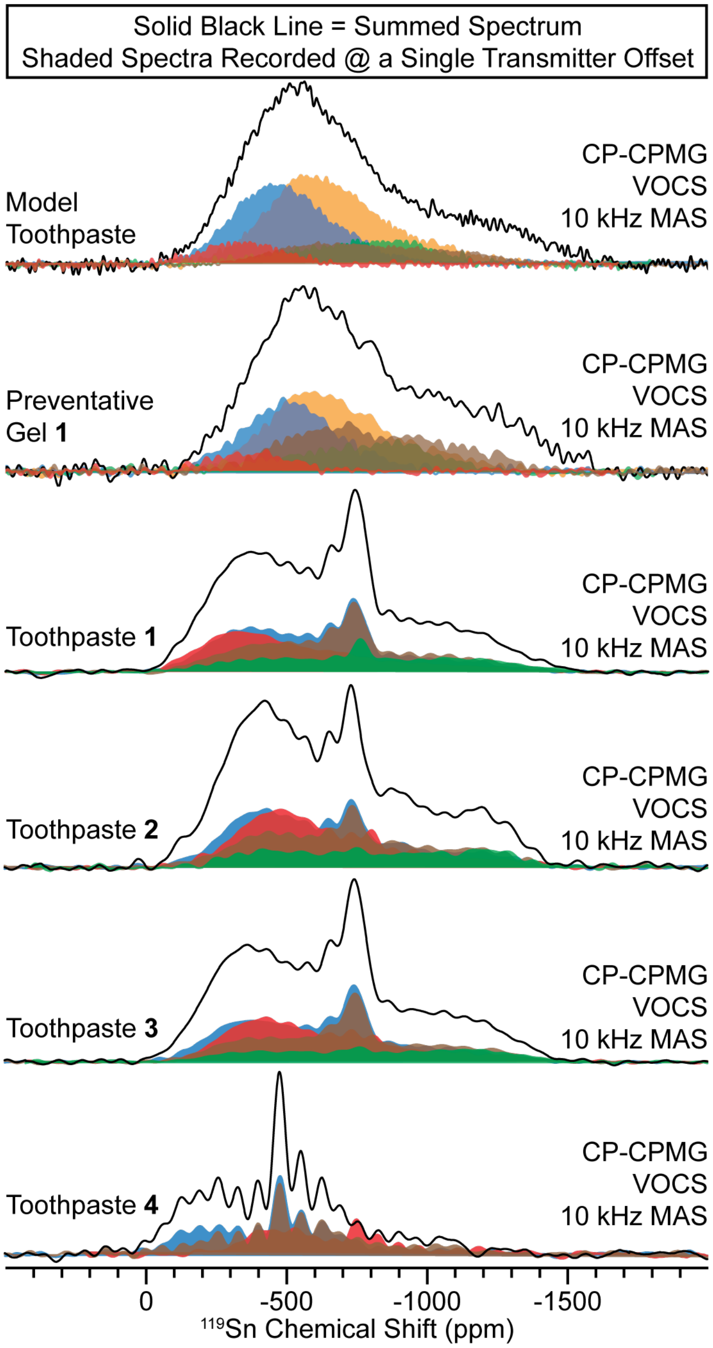

Supplementary Figure 11. DNP-enhanced $^{1}H \rightarrow ^{119}Sn$ CP-CPMG NMR spectra of (upper to lower) the model toothpaste, preventative gel **1**, and toothpastes **1**, **2**, **3** and **4** recorded with a 10 kHz MAS frequency and variable ^{119}Sn transmitter offsets (i.e., VOCS style acquisition). Sub-spectra for specific ^{119}Sn transmitter offsets are shown with different solid colors below the summed NMR spectrum (black trace).

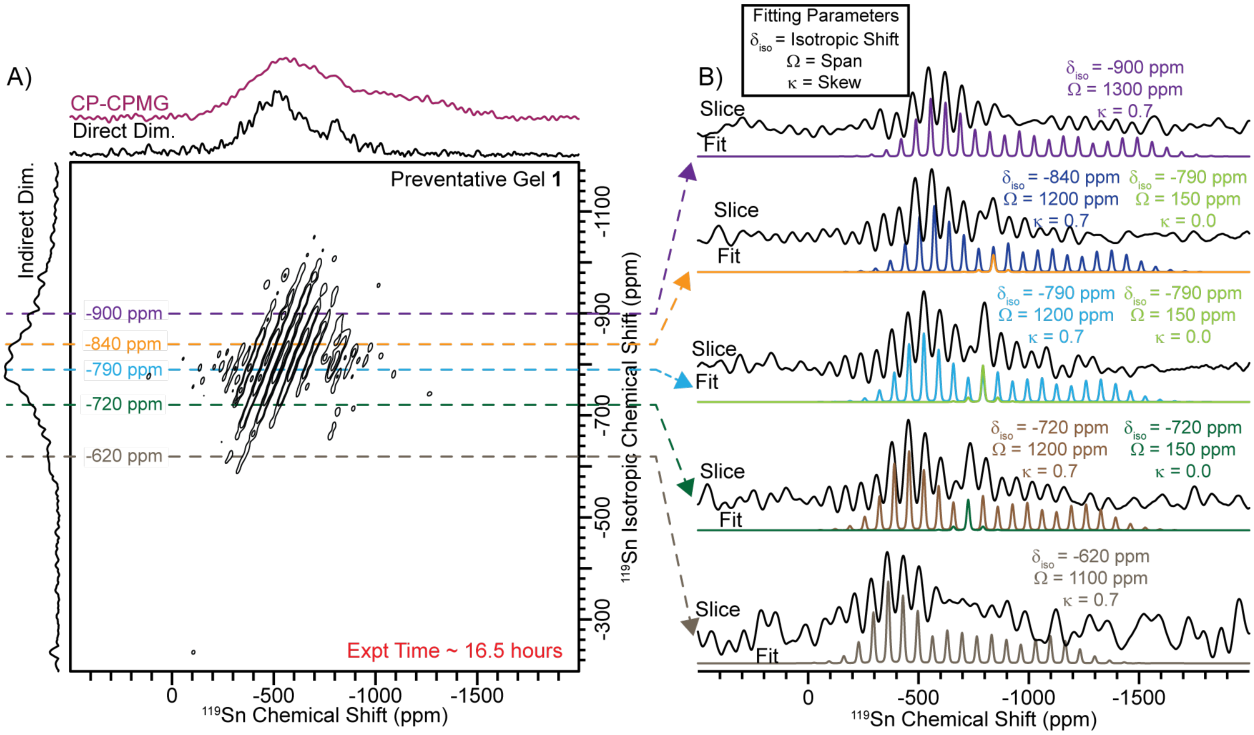

Supplementary Figure 12. (A) DNP-enhanced 2D ^{119}Sn aMAT NMR spectrum of preventative gel **1** acquired with a 10 kHz MAS frequency, ^{1}H→^{119}Sn CP at the start of the experiment, and CPMG for ^{119}Sn detection. (B) ^{119}Sn NMR spectra extracted from the 2D aMAT NMR spectrum at the indicated ^{119}Sn isotropic chemical shifts (δ_{iso}). Analytically simulated spectra are shown (colored traces) below the (black trace) experimental MAS spectra. For some rows, multiple sites were used during fitting.

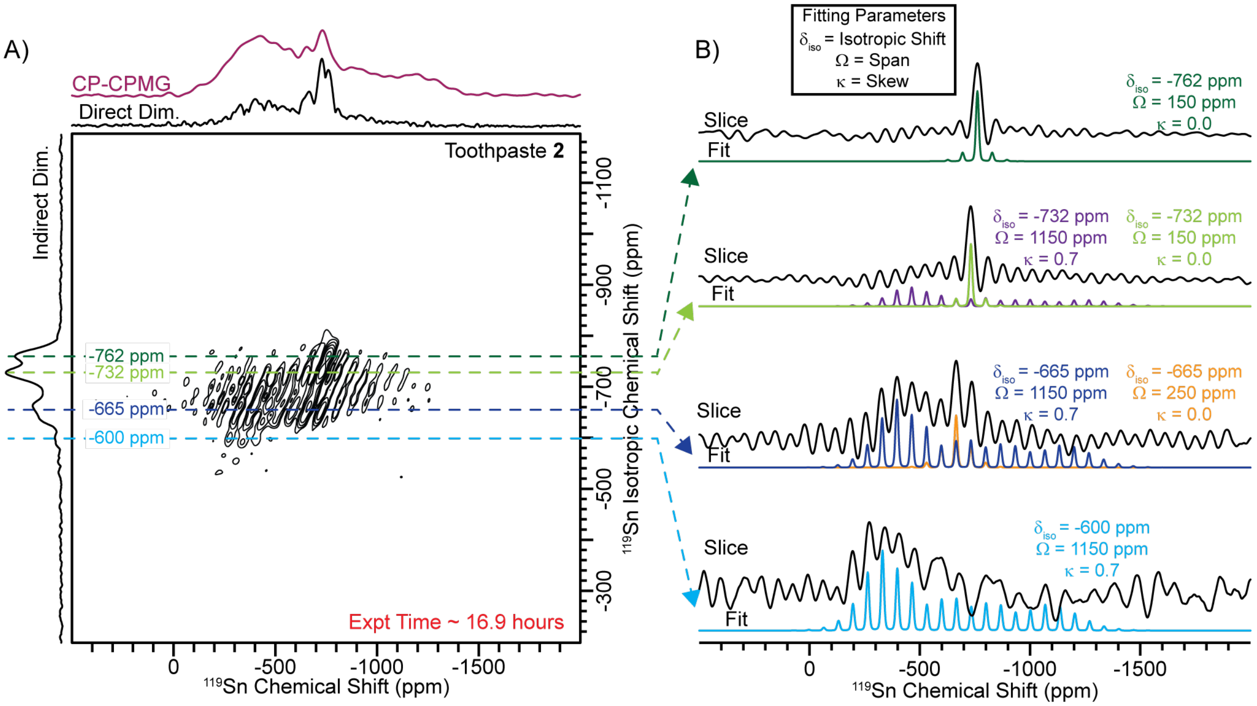

Supplementary Figure 13. (A) DNP-enhanced 2D ^{119}Sn aMAT NMR spectrum of toothpaste **2** acquired with a 10 kHz MAS frequency, $^{1}H \rightarrow ^{119}Sn$ CP at the start of the experiment, and CPMG for ^{119}Sn detection. (B) ^{119}Sn NMR spectra extracted from the 2D aMAT NMR spectrum at the indicated ^{119}Sn isotropic chemical shifts (δ_{iso}). Analytically simulated spectra are shown (colored) below the (black) experimental MAS spectra.

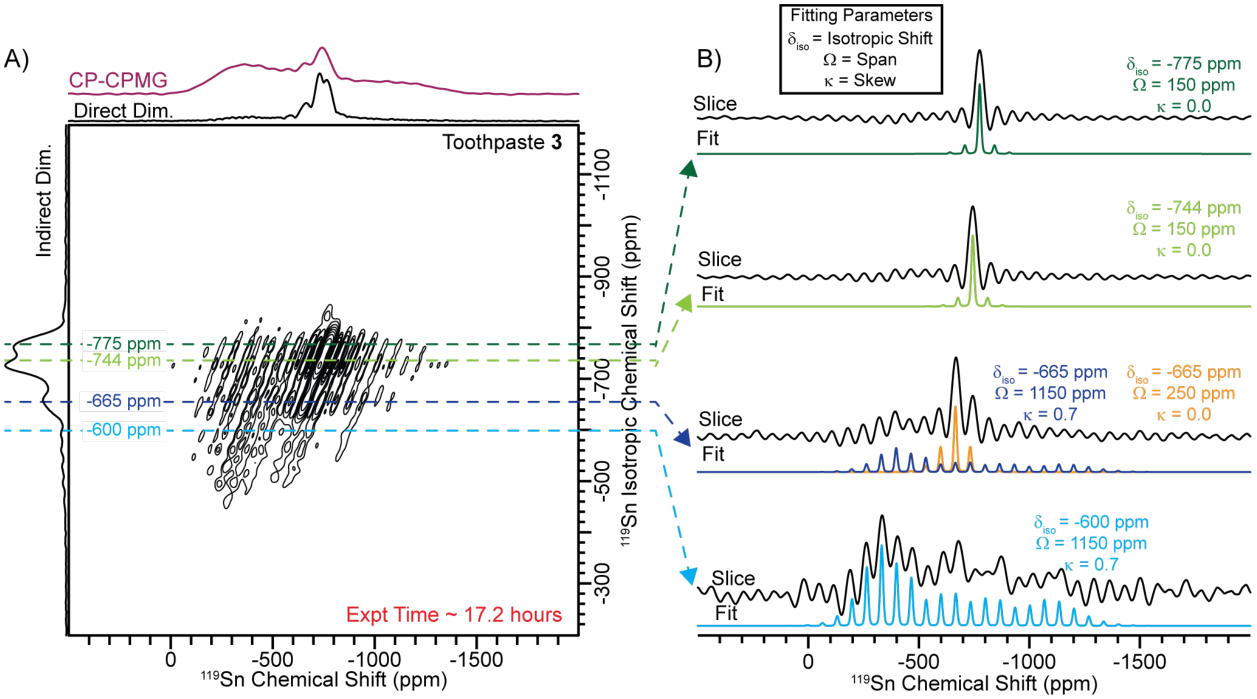

Supplementary Figure 14. (A) DNP-enhanced 2D ^{119}Sn aMAT NMR spectrum of toothpaste **3** acquired with a 10 kHz MAS frequency, ^{1}H→^{119}Sn CP at the start of the experiment, and CPMG for ^{119}Sn detection. (B) ^{119}Sn NMR spectra extracted from the 2D aMAT NMR spectrum at the indicated ^{119}Sn isotropic chemical shifts (δ_{iso}). Analytically simulated spectra are shown (colored) below the (black) experimental MAS spectra.

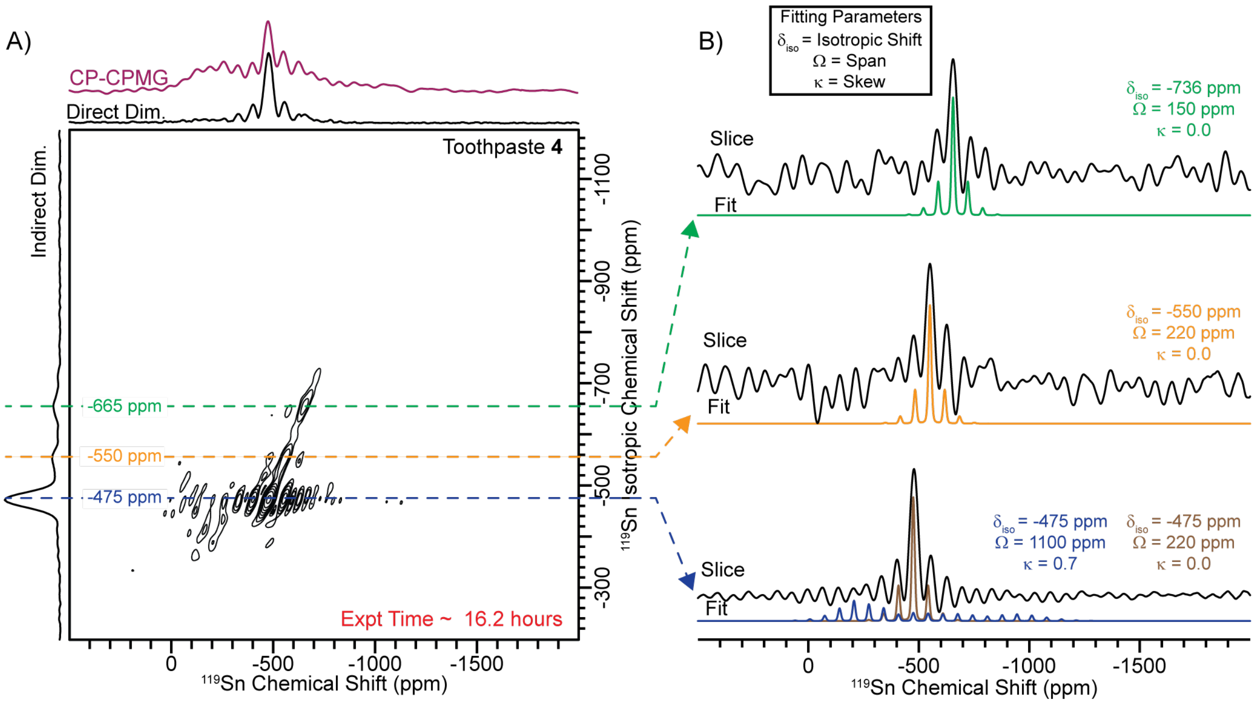

Supplementary Figure 15. (A) DNP-enhanced 2D ^{119}Sn aMAT NMR spectrum of toothpaste **4** acquired with a 10 kHz MAS frequency, ^{1}H→^{119}Sn CP at the start of the experiment, and CPMG for ^{119}Sn detection. (B) ^{119}Sn NMR spectra extracted from the 2D aMAT NMR spectrum at the indicated ^{119}Sn isotropic chemical shifts (δ_{iso}). Analytically simulated spectra are shown (colored) below the (black) experimental MAS spectra.

Supplementary Table 3. ^{119}Sn CP-CPMG NMR Spectral Fitting Parameters of Toothpaste Samples.

Sn Oxidation State	δ_{iso} (ppm)	δ_{iso} distribution (ppm)[b]	Ω (ppm)	κ	Population (%)
		Model Toothpaste			
+4	–675	134	150	0[c]	8
+2	–800	134	1200	0.7	50
+2	–650	134	1200	0.7	42
		Preventative Gel ***1***			
+4	–800	67	150	0	3
+4	–720	67	150	0	4
+2	–820	134	1200	0.7	67
+2	–650	134	1200	0.7	26
		Toothpaste ***1***			
+4	–750	67	150	0[c]	12
+4	–655	67	250	0[c]	8
+2	–630	201	1200	0.7	80
		Toothpaste ***2*** **(Fresh)**			
+4	–738	54	150	0[c]	7
+4	–655	50	250	0[c]	3
+2	–670	201	1150	0.7	90
		Toothpaste ***2*** **(1 Day Air Exposed)**			
+4	–738	54	150	0[c]	10
+4	–655	50	250	0[c]	7
+2	–670	201	1150	0.7	83
		Toothpaste ***3***			
+4	–749	67	150	0[c]	13
+4	–655	54	250	0[c]	7
+2	–630	201	1200	0.7	80
		Toothpaste ***4***			
+4	–639	33	220	0[c]	6
+4	–550	33	220	0[c]	5
+4	–475	33	220	0[c]	21
+2	–475	134	1100	0.7	68

[a]The color of the text for each site corresponds to the color of the fit in Figure 5 of the main text.
[b]The distribution in δ_{iso} was determined from the amount of Gaussian line-broadening used in the fit, where the distribution is equal to ± 1σ (i.e., 68.2 % of the area in the Gaussian curve).
[c]CSA is too small to accurately determine κ.

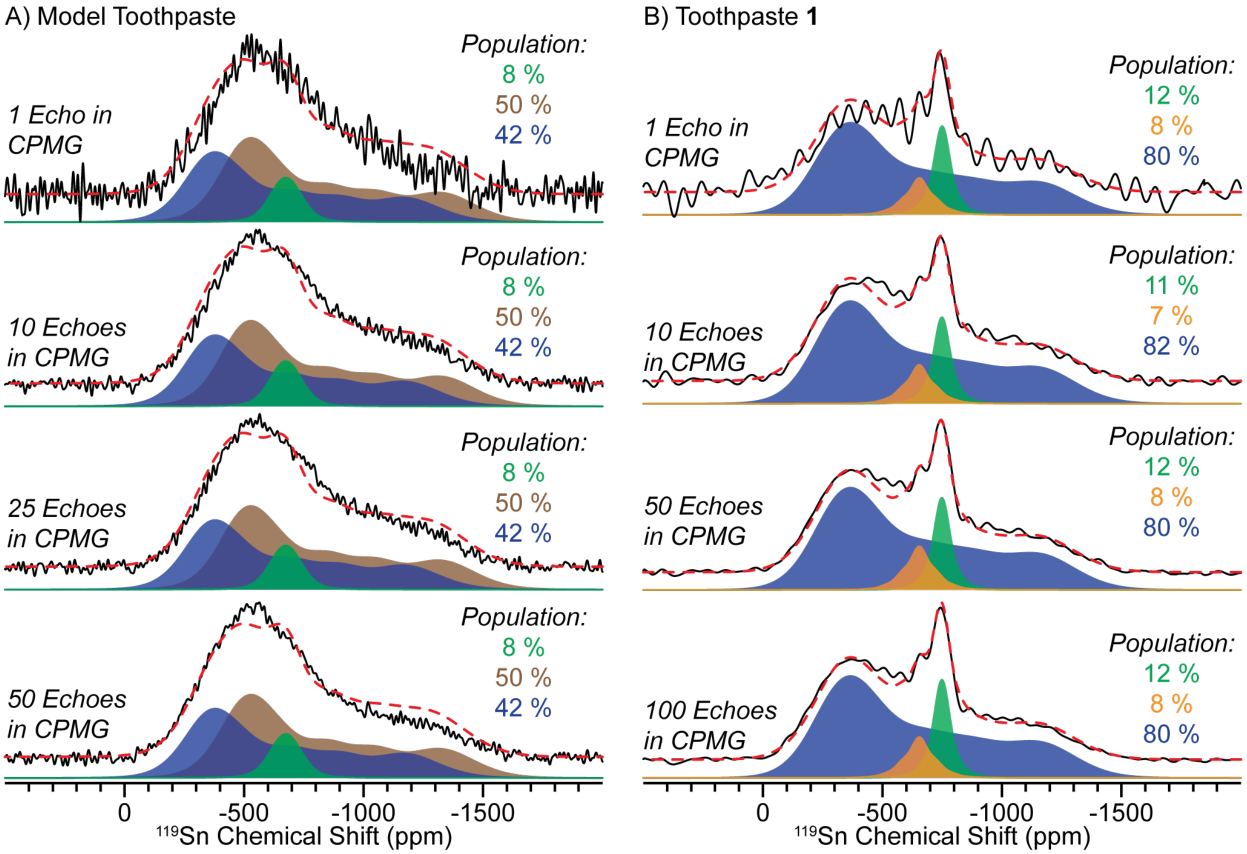

Supplementary Figure 16. DNP-enhanced $^{1}H \rightarrow ^{119}Sn$ CP-CPMG NMR spectra of (A) the model toothpaste and (B) toothpaste **1**. The spectra were processed by using the indicated number of spin echoes within each CPMG train. The total experimental spectrum is shown as the black trace. The total summed fit is shown as a dashed red trace. Solid colored patterns (blue, green, brown and orange) correspond to the individual sites used in the fits. Populations (integrated intensities) for each site are indicated to the right.

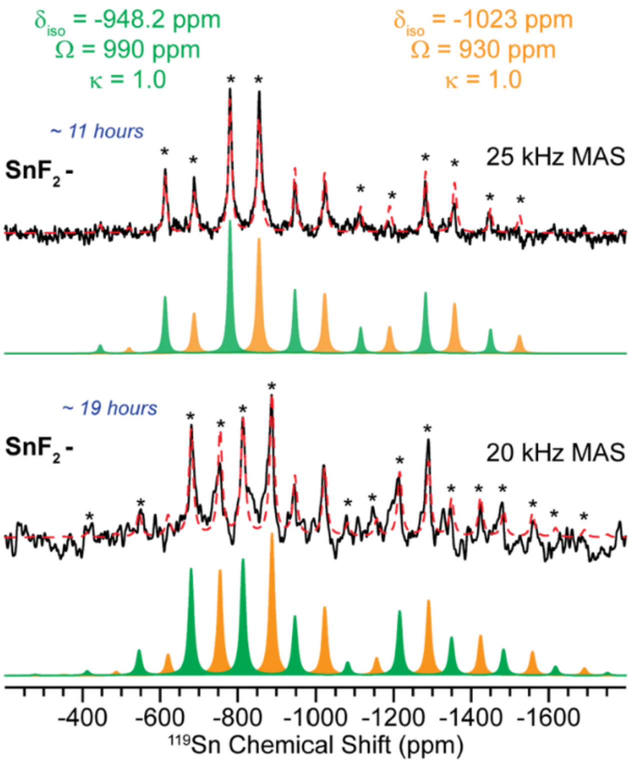

Supplementary Figure 17. ^{119}Sn solid-state NMR spectra of SnF_2 obtained with MAS frequencies of 25 kHz (upper) and 20 kHz (lower) to assign isotropic signals and spinning sidebands. Experimental spectra are shown as black traces, while the total analytical simulation is shown as a dashed red trace. Asterisks denote spinning sidebands. The individual fitted sideband intensities for the SnF_3 and SnF_5 sites are shown as solid green and orange peaks, respectively.

Supplementary Table 4. Experimental NMR parameters.

Figure	Expt.	MAS (kHz)	$\tau_{rec.\ delay}$ (s)	# of Scans	Δt_1 (μs)	t_1 TD Points	t_1 AQ (ms)	$\tau_{CP/J\text{-}evol.}$ (ms)	Total Expt (h)
1B (upper)	^{119}Sn Spin Echo	25	150	400	-	-	-	-	16.7
1B (middle)	^{119}Sn Spin Echo	25	50	848	-	-	-	-	11.8
1B (lower)	^{119}Sn Spin Echo	25	100	384	-	-	-	-	10.7
1C (upper)	2D ^{19}F{^{119}Sn} *J*-HMQC	25	5	32	2.5	256	0.32	0.32[a]	11.4
1C (lower)	2D ^{19}F{^{119}Sn} *J*-HMQC	20	10	64	4.0	128	0.256	0.1[a]	22.8
2A (upper)	^{19}F Spin Echo	Solution	20[b]	8	-	-	-	-	0.04
2A (middle)	^{19}F Spin Echo	25	30[b]	16	-	-	-	-	0.13
2A (lower)	^{19}F Spin Echo	25	750[c]	4	-	-	-	-	0.83
2C	2D ^{19}F{^{119}Sn} *J*-HSQC	Solution	0.4	8	20	320	3.2	0.28[a]	0.28
3A (MW On)	^{1}H→^{13}C CPMAS	10	20	4	-	-	-	1[d]	0.02
3A (MW Off)	^{1}H→^{13}C CPMAS	10	20	32	-	-	-	1[d]	0.18
3B (MW On)	^{1}H→^{119}Sn CP-CPMG	10	20	4	-	-	-	6[d]	0.02
3B (MW Off)	^{1}H→^{119}Sn CP-CPMG	10	20	128	-	-	-	6[d]	0.71
3C	2D ^{1}H→^{13}C CP-HETCOR	10	2	4	64	256	8.192	1[d]	0.57
3D	2D ^{1}H→^{119}Sn CP-HETCOR	10	20	16	64	128	4.096	6[d]	11.4
4A	2D ^{119}Sn aMAT	10	7.28	14	3.4	200	0.34	2[d]	5.7
4C	2D ^{119}Sn aMAT	10	4	112	3.4	124	0.211	6[d]	15.4
5 (model)	^{1}H→^{119}Sn CP-CPMG	10	20	64 (5)[e]	-	-	-	6[d]	1.8[f]
5 (Gel **1**)	^{1}H→^{119}Sn CP-CPMG	10	15.9	256 (5)[e]	-	-	-	6[d]	5.6[f]
5 (Toothpaste **1**)	^{1}H→^{119}Sn CP-CPMG	10	4	256 (4)[e]	-	-	-	6[d]	1.1[f]
5 (Toothpaste **2**)	^{1}H→^{119}Sn CP-CPMG	10	4	512 (4)[e]	-	-	-	6[d]	2.3[f]
5 (Toothpaste **2** - air)	^{1}H→^{119}Sn CP-CPMG	10	8	384 (4)[e]	-	-	-	6[d]	3.4[f]
5 (Toothpaste **3**)	^{1}H→^{119}Sn CP-CPMG	10	4	256 (4)[e]	-	-	-	6[d]	1.1[f]
5 (Toothpaste **4**)	^{1}H→^{119}Sn CP-CPMG	10	2	1024 (3)[e]	-	-	-	6[d]	1.7[f]
S1	2D ^{19}F{^{119}Sn} *J*-HMQC	25	2	64	10	128	0.64	0.32[a]	4.6
S2 (Supplier **a**)	^{19}F Spin Echo	25	50[b]	16	-	-	-	-	0.22
S2 (Supplier **b** ball milled)	^{19}F Spin Echo	25	100[b]	4	-	-	-	-	0.11
S3	2D ^{19}F{^{119}Sn} *J*-HMQC	25	10	32	4	128	0.256	0.08[a]	11.4
S5	2D ^{19}F{^{119}Sn} *J*-HMQC	Solution	0.5	8	20	160	1.6	1.2[a]	0.18
S6 (upper)	^{119}Sn Spin Echo	Solution	1	128	-	-	-	-	0.04
S7B	^{1}H→^{13}C CPMAS	10	5	4	-	-	-	1[d]	0.01
S8B (MW on)	^{1}H→^{119}Sn CP-CPMG	10	4	256	-	-	-	6[d]	0.28
S8B (MW off)	^{1}H→^{119}Sn CP-CPMG	10	4	1024	-	-	-	6[d]	1.1
S8C (MW on)	^{1}H→^{119}Sn CP-CPMG	10	4	512	-	-	-	6[d]	0.57

S8C (MW off)	$^1H \rightarrow ^{119}Sn$ CP-CPMG	10	4	1088	-	-	-	6^d	1.2
S8D (MW on)	$^1H \rightarrow ^{119}Sn$ CP-CPMG	10	4	64	-	-	-	6^d	0.07
S8D (MW off)	$^1H \rightarrow ^{119}Sn$ CP-CPMG	10	4	784	-	-	-	6^d	0.87
S8E (MW on)	$^1H \rightarrow ^{119}Sn$ CP-CPMG	10	2	1024	-	-	-	6^d	0.57
S8E (MW off)	$^1H \rightarrow ^{119}Sn$ CP-CPMG	10	2	2048	-	-	-	6^d	1.1
S9A	2D $^1H \rightarrow ^{13}C$ CP-HETCOR	10	2	4	64	256	8.192	1^d	0.57
S9B	2D $^1H \rightarrow ^{119}Sn$ CP-HETCOR	10	15.9	32	64	100	3.2	6^d	14.1
S12	2D ^{119}Sn aMAT	10	10.4	42	3.4	136	0.232	2^d	16.5
S13	2D ^{119}Sn aMAT	10	4	112	3.6	136	0.245	6^d	16.9
S14	2D ^{119}Sn aMAT	10	4	112	3.6	138	0.248	6^d	17.2
S15	2D ^{119}Sn aMAT	10	2	280	3.4	104	0.177	6^d	16.2

[a] *J*-evolution duration. [b] Quantitative recycle delay ($\geq 5 \times T_1$). [c] Recycle delay *ca.* 4-5 $\times T_1$. [a] CP contact time. [e] Number of scans for each transmitter offset. The total number of ^{119}Sn offsets used to construct the VOCS spectrum is given in the parenthesis. [f] Total experimental time for all ^{119}Sn transmitter offsets.
